# Urinary Glyphosate Concentrations and Serum Sex Hormones in a Nationally Representative U.S. Sample: NHANES 2017–2018

**DOI:** 10.3390/life15071024

**Published:** 2025-06-27

**Authors:** Wen-Yang Wu, Du-Sheng Wang, Hsuan-Cheng Lin, Chikang Wang, Chien-Yu Lin

**Affiliations:** 1 School of Medicine, National Taiwan University, Taipei City 100, Taiwan; b09401017@ntu.edu.tw; 2 Department of Obstetrics and Gynecology, En Chu Kong Hospital, New Taipei City 237, Taiwan; 00012@km.eck.org.tw; 3 School of Medicine, College of Medicine, Taipei Medical University, Taipei City 110, Taiwan; b101114002@tmu.edu.tw; 4 Department of Environmental Engineering and Health, Yuanpei University of Medical Technology, Hsinchu City 300, Taiwan; ckwang@mail.ypu.edu.tw; 5 Department of Internal Medicine, En Chu Kong Hospital, New Taipei City 237, Taiwan; 6 School of Medicine, College of Medicine, Fu Jen Catholic University, New Taipei City 242, Taiwan

**Keywords:** androstenedione, estradiol, glyphosate, glyphosate-based herbicides (GBHs), serum sex hormones, National Health and Nutrition Examination Survey (NHANES)

## Abstract

Glyphosate and glyphosate-based herbicides (GBHS) are the most widely used herbicides worldwide, yet their potential endocrine-disrupting effects in humans remain inadequately studied. We analyzed data from 1532 participants aged ≥6 years in the 2017–2018 National Health and Nutrition Examination Survey (NHANES). Serum sex hormones assessed included follicle-stimulating hormone (FSH), luteinizing hormone (LH), anti-Müllerian hormone (AMH), androstenedione, estrone, estradiol, estrone sulfate, 17α-hydroxyprogesterone, progesterone, and sex hormone-binding globulin (SHBG). We found that higher urinary glyphosate levels were significantly associated with lower concentrations of AMH (β = −0.140, *p* < 0.05), androstenedione (β = −0.134, *p* < 0.001), estradiol (β = −0.185, *p* < 0.05), estrone (β = −0.132, *p* < 0.05), estrone sulfate (β = −0.196, *p* < 0.001), 17α-hydroxyprogesterone (β = −0.097, *p* < 0.05), and progesterone (β = −0.212, *p* < 0.05). SHBG was positively associated (β = 0.080, *p* < 0.05). FSH and LH showed no significant associations. These associations were generally linear and showed modification by age. Subgroup analyses revealed stronger negative associations in adult males, while SHBG increased in females. In conclusion, we observed that higher urinary glyphosate levels were significantly associated with alterations in multiple serum sex hormones. Although the cross-sectional design precludes causal inference, these findings underscore the need for longitudinal research to determine temporal relationships and underlying mechanisms.

## 1. Introduction

Glyphosate and glyphosate-based herbicides (GBHs), first introduced for commercial use in 1974, have become the most widely used herbicides globally, prized for their effectiveness against a broad range of weeds and their perceived safety [1]. The arrival of genetically engineered crops designed to tolerate glyphosate in 1996 significantly expanded their application in farming [1]. Analysts predict that the global herbicide market will grow from USD 11.36 billion in 2024 to USD 26.39 billion by 2037, with annual growth exceeding 6.7% [2]. However, the detection of these herbicides in various foods and even in humans has raised concerns regarding their potential impact on both the environment and people’s well-being [3,4]. Research highlighting the potential of glyphosate to harm cells and DNA has led the International Agency for Research on Cancer to designate it as a likely carcinogen for humans [5]. On the other hand, organizations such as the U.S. Environmental Protection Agency and the European Food Safety Authority argue that glyphosate poses little cancer risk when applied according to recommended standards [6,7]. This disagreement among experts has prompted diverse regulatory responses across the globe. For instance, Germany imposed a total ban on glyphosate and GBHs in 2024 [8], while the United States has not yet prohibited their use, though they have faced costly legal battles resulting in large settlements tied to claims linking glyphosate and GBHs to non-Hodgkin lymphoma [9].

There are growing concerns about glyphosate’s potential to disrupt endocrine function, particularly in relation to sex hormones. In vitro studies show that glyphosate can mimic estrogen and interfere with hormone receptor activity [10,11]. Animal studies report that exposure to glyphosate or GBHs affects sex hormone levels and reproductive development, with outcomes depending on timing and dosage [12,13,14]. However, to date, no human occupational studies have directly assessed the relationship between glyphosate exposure and sex hormone levels. This gap is notable given that occupationally exposed individuals may experience higher and more prolonged exposure levels compared to the general population. Existing research has focused on the general population, particularly through analyses of National Health and Nutrition Examination Survey (NHANES) data. However, these studies have examined only a narrow range of hormones—primarily testosterone, estradiol, and sex hormone-binding globulin (SHBG). Findings suggest that higher urinary glyphosate levels may be associated with lower estradiol and testosterone concentrations [15,16].

Since the relationship between glyphosate exposure and sex hormones has not been comprehensively explored in previous epidemiological studies, this represents a significant research gap. Gaining a deeper understanding of how glyphosate influences sex hormone regulation in humans is essential, given its potential implications for reproductive and endocrine health. To address this gap, we analyzed data from the 2017–2018 cycle of the NHANES, which includes measurements of urinary glyphosate levels and a broad panel of sex hormone biomarkers: follicle-stimulating hormone (FSH), luteinizing hormone (LH), anti-Müllerian hormone (AMH), androstenedione, estrone, estradiol, estrone sulfate, 17α-hydroxyprogesterone, progesterone, and SHBG. Our study is the first to provide epidemiological evidence linking glyphosate exposure to a comprehensive set of sex hormone biomarkers in a nationally representative U.S. population.

## 2. Materials and Methods

### 2.1. Study Population

**Figure 1:**
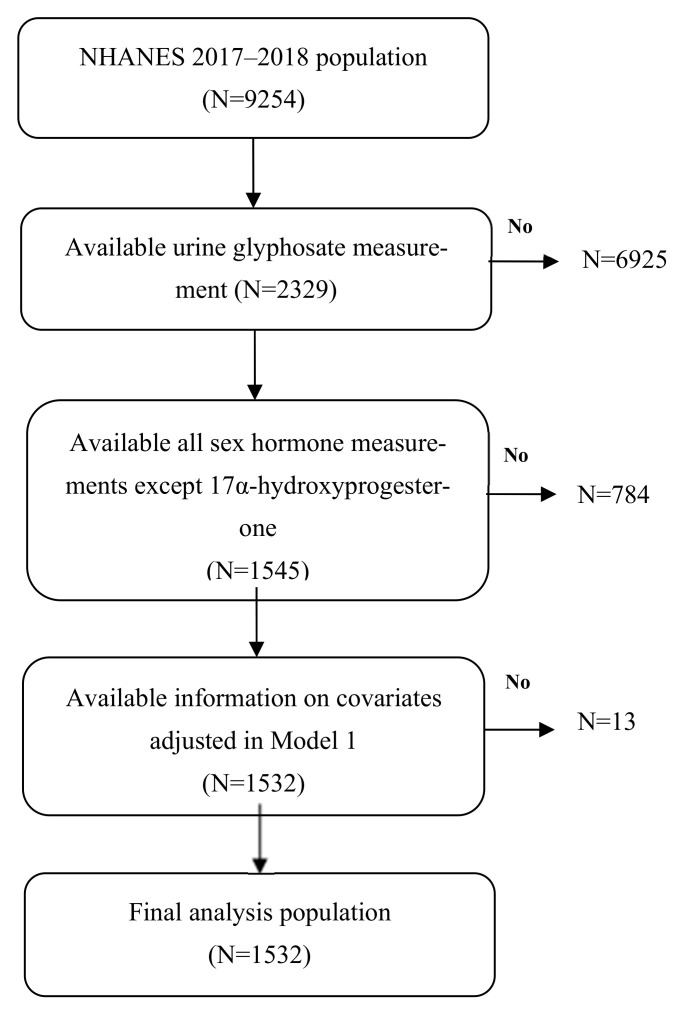
Flow chart algorithm.

The NHANES, carried out every two years, aims to offer a comprehensive overview of the health and nutritional status of the civilian, non-institutionalized population across the United States. This survey utilizes an intricate, multi-phase probability sampling approach to guarantee that its results reflect the broader U.S. populace. Extensive details regarding the survey’s methodology and consent processes are accessible on the NHANES website [17]. In this study, we examined data from the 2017–2018 NHANES cycle, which initially encompassed 9254 participants. Among them, 2329 individuals provided urine samples for glyphosate analysis, while 1545 had complete data for all sex hormone measurements, excluding 17α-hydroxyprogesterone. After applying the necessary covariate criteria, 1532 participants qualified for inclusion in Model 1 of our multiple regression analysis. Participants with missing values for any exposure, outcome, or covariate were excluded from the analysis; no imputation was performed. The participant selection procedure is depicted in Figure 1.

### 2.2. Measurement of Urinary Glyphosate Levels

In the 2017–2018 NHANES survey, urinary glyphosate levels were assessed for all participants aged 3 to 5 years and for a randomly selected one-third subset of individuals aged 6 years and older. This study focused on participants aged 6 and above. In brief, the measurement of urinary glyphosate concentrations was conducted using a sophisticated two-dimensional online ion chromatography system paired with tandem mass spectrometry (IC-MS/MS), alongside isotope dilution quantification. The IC-MS/MS setup was calibrated using standard glyphosate solutions, and its accuracy was confirmed by analyzing quality control samples of both high and low concentrations during each run. These control results were compiled, averaged, and subjected to standard statistical analysis to verify the precision and trustworthiness of the data. For the 2017–2018 NHANES cycle, the lower limit of detection (LOD) for glyphosate was established at 0.1 ng/mL. Any values falling below this threshold were estimated by dividing the LOD by the square root of 2. Comprehensive details of the analytical methods used can be found on the NHANES website [18].

### 2.3. Measurement of Serum Sex Hormone Levels

During the 2017–2018 NHANES cycle, serum sex hormones assessed include FSH, LH, AMH, androstenedione, estrone, estradiol, estrone sulfate, 17α-hydroxyprogesterone, progesterone, and SHBG. These hormones are quantified using isotope dilution liquid chromatography tandem mass spectrometry for high accuracy. Samples are collected from participants aged 6 years and older, with eligibility for 17α-hydroxyprogesterone testing beginning at age 13. Detailed analytical procedures are outlined on the NHANES website [19].

The hormones and their origins are as follows: FSH, secreted by the anterior pituitary, promotes ovarian follicle growth in females to produce estrogen and aids spermatogenesis in males through Sertoli cell action. LH, also from the anterior pituitary, induces ovulation in females via follicle rupture and boosts testosterone synthesis in males through Leydig cells [20]. AMH, produced by ovarian granulosa cells in females as an indicator of ovarian reserve, and by Sertoli cells in males, serves as a marker of Sertoli cell function with potential roles in testicular regulation and fertility evaluation [20]. Androstenedione, from adrenals and ovaries (females) or testes (males), acts as a precursor to testosterone and estrone [21]. Estrone, estradiol, and estrone sulfate are estrogens with varying potency and sources: estrone is a weaker estrogen mainly from peripheral aromatization; estradiol is the most potent, primarily from ovarian follicles and testes; and estrone sulfate serves as an inactive reservoir, converted to active forms as needed [22]. 17α-hydroxyprogesterone, from adrenals and ovaries, functions as a progesterone precursor. Progesterone, from ovaries and corpus luteum, maintains pregnancy and, in non-pregnant states, prepares the uterus for implantation and regulates menstruation. SHBG, from the liver, binds androstenedione and estradiol with strong affinity [23].

### 2.4. Covariates

Information on sociodemographic characteristics, including age, gender, and ethnicity, was obtained from the NHANES database. Because Tanner staging is restricted in NHANES 2013–2018, we used nationally accepted reference ages for puberty onset—11 years for girls (breast Tanner II) and 12 years for boys (testicular volume ≥ 4 mL)—to distinguish pre-pubertal from pubertal participants [24]. We also used 18 years as the threshold for post-pubertal status, as U.S. longitudinal data show that more than 97% of both girls and boys have reached Tanner V and adult hormone levels by this age [25]. Adult females were classified as post-menopausal if they reported “menopause/change of life” as the reason for 12 months of amenorrhea or indicated that both ovaries had been removed. For those who reported hysterectomy or had missing questionnaire data, post-menopause was assigned when serum estradiol was <30 pg/m [26]. BMI z-scores were determined using age-specific methods. For individuals aged 6 to 19 years, BMI-for-age z-scores were computed using the CDC 2000 growth charts and the LMS method, which incorporates age- and sex-specific distributional parameters [27]. For those aged 20 years and above, BMI z-scores were calculated by taking the difference between an individual’s BMI and the average BMI of the NHANES 2017–2018 adult reference population, then dividing by the standard deviation of BMI within that reference group. This approach enables the comparison of BMI values in relation to the distribution of the current U.S. adult population. Smoking status was classified into three categories—current smokers, those exposed to environmental tobacco smoke (ETS), and non-smokers—based on responses to the NHANES smoking questionnaire [28]. Alcohol use was evaluated by asking participants if they had consumed at least 12 alcoholic beverages in the previous year. Physical activity levels were determined by combining activity scores with their corresponding metabolic equivalent of task values, as outlined in the NHANES website guidelines [29]. High-sensitivity C-reactive protein (hs-CRP) was measured in serum using latex-enhanced nephelometry. Estimated glomerular filtration rate (eGFR) was estimated using the 2021 creatinine equation developed by the Chronic Kidney Disease Epidemiology Collaboration [30]. Urinary creatinine was treated as an independent variable in this analysis, without adjustments for hydration status.

### 2.5. Statistics

We used sampling weights from NHANES’s multistage stratified design to ensure national representativeness, applying them via SPSS Complex Samples to obtain accurate standard errors and significance tests [31]. Random effects were not applied, as variance estimation was based on Taylor series linearization, which accounts for clustering and stratification inherent in the NHANES complex sampling design. To compare urinary creatinine-adjusted glyphosate concentrations across demographic and behavioral subgroups, we conducted two-tailed Student’s *t*-test for binary variables (e.g., sex, smoking status) and one-way analysis of variance for variables with more than two categories (e.g., age group, ethnicity, BMI z-score, alcohol consumption). To explore the association between urinary glyphosate levels and serum sex hormones, we performed linear regression analyses tailored for complex sampling designs. In Model 1, we adjusted for age, gender, ethnicity, BMI z-scores, and urinary creatinine, all of which are standard confounders in endocrine-related environmental health studies. To investigate possible non-linear patterns, we conducted supplementary analyses by adding polynomial terms. To explore potential effect modification, we tested interaction terms between urinary glyphosate concentrations and key covariates known to influence hormone regulation: age, sex, race/ethnicity, and BMI z-score. These interaction terms (e.g., glyphosate × age, glyphosate × sex) were included one at a time in the survey-weighted linear regression models based on Model 1 covariates. This approach allowed us to assess whether the association between glyphosate exposure and hormone levels varied significantly across demographic and physiological subgroups. For trend analysis, glyphosate exposure quartiles were modeled as an ordinal continuous variable in the survey-weighted linear regression. The *p* for trend was determined by using the Wald test for the regression coefficient, accounting for the complex sampling design.

Given the strong influence of age and sex on hormone levels, we conducted sensitivity analyses by stratifying participants into four groups based on sex (male and female) and age (<18 and ≥18 years). Among participants under 18 years, we further stratified by developmental stage (children vs. adolescents) to explore age-specific associations between urinary glyphosate and serum sex hormones. However, due to the limited number of participants in this age group (N = 223; 39 children and 184 adolescents, including 96 boys and 127 girls), sex-specific analyses were not performed within these subgroups in order to preserve statistical power in the complex sample regression models. Among adult females, we also stratified by menopausal status to account for major hormonal changes associated with reproductive aging. To further examine associations in adults (≥18 years), we constructed Model 2, which included additional adjustments for smoking status, alcohol consumption, eGFR, and physical activity—covariates available only in the adult NHANES dataset and selected to account for behavioral, metabolic, and renal factors that may influence both urinary glyphosate levels and endogenous hormone concentrations. We additionally conducted sensitivity analyses by including hs-CRP as a covariate to account for potential confounding from systemic inflammation. The normality of continuous covariates was evaluated using the Shapiro–Wilk test. Due to their non-Gaussian distribution, the natural logarithms (ln) of glyphosate, sex hormones, urinary creatinine, eGFR, and physical activity were employed in the analysis. All statistical analyses were performed using SPSS version 30 (SPSS Inc., Chicago, IL, USA), with a significance level set at *p* < 0.05. *p* values are shown to three decimal places when ≥0.05 and as thresholds when <0.05 (*p* < 0.05, *p* < 0.01, *p* < 0.001).

## 3. Results

The participants in this study had an average age of 44.17 years (SD = 21.30), with ages spanning from 6 to 80 years. Detectable glyphosate levels were observed in about 81.1% of the individuals. Table 1 displays the average urinary glyphosate concentrations (adjusted for urinary creatinine) and their standard deviations across various demographic categories. Females exhibited higher glyphosate levels than males, while participants aged 60 and older showed the highest concentrations, followed by those aged 3–17, with the lowest levels found in the 18–59 age range. Among ethnic groups, non-Hispanic whites recorded the highest glyphosate levels. Individuals with lower BMI z-scores had elevated glyphosate concentrations, which tended to decrease as BMI z-scores rose. Among those aged 18 and older, non-smokers displayed higher glyphosate levels compared to current smokers and those exposed to ETS. Furthermore, participants consuming fewer than 12 alcoholic drinks annually had slightly lower glyphosate levels than those with higher alcohol intake. Table 2 presents the median and interquartile range (25th–75th percentile) of urinary glyphosate and serum sex hormone levels stratified by age group (children, adolescents, and adults), with adult females further categorized into pre- and post-menopausal groups. Supplementary Table S1 compares serum sex hormone concentrations in participants with detectable versus undetectable urinary glyphosate. Those with detectable glyphosate showed a modestly lower mean FSH level (17.81 mIU/mL vs. 21.63 mIU/mL; *p* < 0.05), whereas no significant differences were observed for the other hormones.

Table 3 presents the results of linear regression analyses examining the relationship between ln-transformed urinary glyphosate concentrations (in µg/L) and ln-transformed serum sex hormone levels, with coefficients (β) and standard errors (S.E.) weighted to reflect the NHANES sampling strategy, adjusted for age, sex, race/ethnicity, BMI z-score, and urinary creatinine (Model 1). Significant negative associations were found for AMH (β = −0.140, *p* < 0.05), androstenedione (β = −0.134, *p* < 0.001), estradiol (β = −0.185, *p* < 0.05), estrone (β = −0.132, *p* < 0.05), estrone sulfate (β = −0.196, *p* < 0.001), 17α-hydroxyprogesterone (β = −0.097, *p* < 0.05; n = 1464), and progesterone (β = −0.212, *p* < 0.05). SHBG showed a positive association (β = 0.080, *p* = 0.002). FSH and LH were non-significant. Interaction terms with age, sex, ethnicity, and BMI z-score were tested. Notable interactions emerged between age and the relationship of urinary glyphosate with AMH (β for interaction term = 0.005, *p* < 0.05) and estrone (β for interaction term = 0.003, *p* < 0.05). To explore potential non-linear associations, we performed additional analyses by including squared and cubic terms in the models. These analyses revealed no significant relationships between glyphosate exposure and serum sex hormone levels for either the squared or cubic terms, indicating that the relationship between glyphosate exposure and serum sex hormones is likely linear in nature.

**Figure 2:**
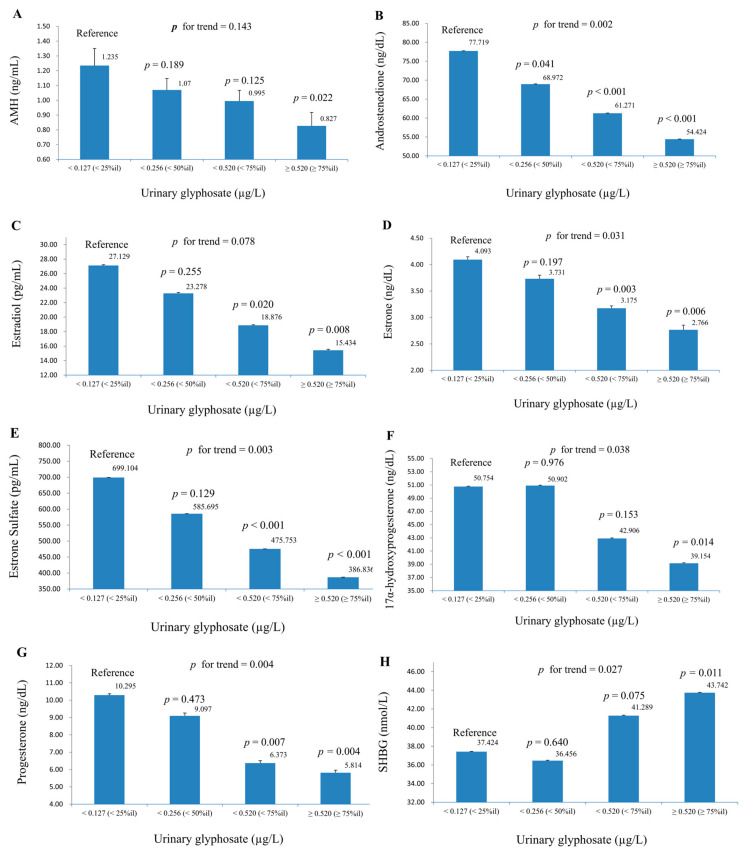
Geometric mean (S.E.) of serum sex hormones across quartiles of urine glyphosate in multiple linear regression models (adjusted for Model 2), with results weighted for sampling strategy. (**A**) AMH. (**B**) Androstenedione. (**C**) Estradiol. (**D**) Estrone. (**E**) Estrone sulfate. (**F**) 17α-hydroxyprogesterone. (**G**) Progesterone. (**H**) SHBG. *p* for trends was calculated by modeling quartiles as an ordinal variable in survey-weighted linear regression, with statistical significance assessed using the Wald test.

Figure 2A–H illustrates the relationship between urinary glyphosate concentrations and various serum sex hormone levels across four glyphosate exposure quartiles. Each panel compares hormone levels to the reference group, with *p* values for individual comparisons and overall trends. For AMH (panel A), levels decreased with higher glyphosate exposure, from 1.335 mIU/mL in the reference group to 0.827 mIU/mL in the highest quartile (*p* = 0.022), though the trend was not significant (*p* for trend = 0.143). Androstenedione (panel B) showed a significant decline from 77.719 ng/dL to 54.424 ng/dL (*p* < 0.001) in the highest quartile, with a strong trend (*p* for trend = 0.002). Estradiol (panel C) decreased from 27.129 pg/mL to 15.443 pg/mL (*p* < 0.001), with a non-significant trend (*p* for trend = 0.078). Estrone (panel D) dropped from 4.093 ng/dL to 2.766 ng/dL (*p* = 0.006), with a non-significant trend (*p* for trend = 0.031). Estrone sulfate (panel E) exhibited a significant reduction from 699.104 pg/mL to 386.836 pg/mL (*p* < 0.001), with a strong trend (*p* for trend = 0.003). 17α-hydroxyprogesterone (panel F) decreased from 50.754 ng/dL to 39.154 ng/dL (*p* = 0.014), but the trend was not significant (*p* for trend = 0.038). Progesterone (panel G) fell from 9.295 ng/dL to 5.814 ng/dL (*p* = 0.004), with a significant trend (*p* for trend = 0.004). SHBG (panel H) showed a slight, non-significant increase from 37.424 nmol/L to 41.429 nmol/L (*p* = 0.011), with no clear trend (*p* for trend = 0.227). Overall, higher urinary glyphosate levels were associated with significant reductions in most sex hormone levels, except for SHBG, which showed a modest increase. The *p* for trends were significant for androstenedione, estrone, estrone sulfate, 17α-hydroxyprogesterone, progesterone, and SHBG.

Table 4 displays a sensitivity analysis for participants under 18, stratified by sex, using NHANES sampling weights and adjusted for Model 1 covariates. No significant associations were found in the overall group. In males, significant effects were observed for estradiol (β = −0.262, *p* < 0.05), estrone sulfate (β = −0.374, *p* < 0.01), and SHBG (β = 0.160, *p* < 0.05). In females, no significant associations emerged. Supplementary Table S2 shows the associations between urinary glyphosate levels and serum sex hormone concentrations among participants under 18 years, stratified into children and adolescents. While most associations were not statistically significant, a few trends were noted. In children, higher glyphosate levels were associated with higher SHBG levels (*p* < 0.05). Table 5 presents a sensitivity analysis for participants aged 18 and older, categorized by sex, with adjustments applied in Model 1 and Model 2. The analysis found no notable links between glyphosate exposure and FSH, LH, AMH, or progesterone levels across any group. However, significant negative correlations were observed in males for androstenedione (Model 2: β = −0.081, *p* < 0.05), estradiol (β = −0.067, *p* < 0.05), estrone (β = −0.088, *p* < 0.01), estrone sulfate (β = −0.094, *p* < 0.05), and 17α-hydroxyprogesterone (β = −0.131, *p* < 0.01), as well as in the overall population for androstenedione (β = −0.069, *p* < 0.05) and 17α-hydroxyprogesterone (β = −0.088, *p* < 0.05). In contrast, SHBG exhibited a significant positive correlation in the total population (β = 0.056, *p* < 0.05) and in females (β = 0.073, *p* < 0.05). Supplementary Table S3 presents associations between urinary glyphosate and serum sex hormones in females aged 18 and older stratified by menopausal status. In pre-menopausal females, no statistically significant associations were observed. In post-menopausal females, higher glyphosate levels were significantly associated with lower estradiol and progesterone concentrations in both models. In Supplementary Table S4, we performed sensitivity analyses by additionally adjusting for hs-CRP in the multivariable models. The inclusion of hs-CRP did not materially change the associations between urinary glyphosate and serum sex hormone concentrations.

## 4. Discussion

While previous research using NHANES data has primarily focused on a limited set of sex hormones—mainly testosterone, estradiol, and SHBG—our study broadens the scope by examining a comprehensive panel of sex hormone biomarkers. We observed that higher urinary glyphosate concentrations were associated with lower serum levels of several sex hormones, including AMH, androstenedione, estradiol, estrone, estrone sulfate, 17α-hydroxyprogesterone, and progesterone, while SHBG showed a positive association. These relationships appeared linear, with no significant non-linear effects detected. Age significantly modified the associations with AMH and estrone. In participants under 18, associations were limited and mostly observed in males. Among adults, glyphosate exposure was linked to hormone reductions primarily in males, whereas females showed increased SHBG levels, suggesting sex-specific hormonal effects. Our findings suggest that glyphosate may have more extensive endocrine-disrupting effects than previously recognized. This broader hormonal disruption, particularly in age- and sex-specific patterns, implies potential risks to reproductive development, hormonal regulation, and long-term health.

Our research showed that urinary creatinine-corrected glyphosate concentrations were notably elevated in females, older adults, non-Hispanic whites, individuals with lower BMI z-scores, non-smokers, and those consuming more than 12 alcoholic drinks annually. Prior research has highlighted dietary intake as a key source of glyphosate exposure [32]. This suggests that individuals with higher glyphosate levels may be regularly consuming foods contaminated with the herbicide due to its widespread use. However, we also observed large standard deviations—especially among females, participants aged 60 and older, and those with lower BMI—indicating high variability within these groups. This variability may limit the consistency and generalizability of our findings.

Studies on glyphosate and sex hormone regulation have shown mixed results, often suggesting possible endocrine-disrupting effects linked to oxidative stress. In vitro experiments have demonstrated that low-dose glyphosate exposure suppresses testosterone biosynthesis in mouse Leydig cells by inducing excessive mitochondrial reactive oxygen species production, leading to mitochondrial dysfunction [33]. Similarly, in human breast cancer cell lines, glyphosate at environmentally relevant concentrations has been shown to emulate estrogen activity by activating estrogen receptor alpha, thereby promoting cellular proliferation [10]. In HepG2 liver cells, higher glyphosate concentrations disrupted aromatase activity and inhibited the transcriptional activity of estrogen and androgen receptors [11]. Animal model studies further revealed that glyphosate and GBHs exerted variable effects on sex hormone levels and reproductive processes. For instance, in male rats, pre-pubertal exposure to GBHs was found to impair reproductive development by reducing testosterone concentrations, delaying puberty onset, and altering seminiferous tubule morphology [12]. In contrast, another study reported that perinatal maternal exposure to glyphosate increased testosterone, estradiol, and sperm production in male offspring, alongside an earlier onset of puberty, effects attributed to disruptions in the hypothalamic–pituitary–gonadal (HPG) axis [13]. These divergent results highlight the significance of exposure timing, suggesting that perinatal exposure may enhance certain reproductive parameters, possibly due to impaired negative feedback mechanisms regulating LH production [13]. In female rats, prenatal glyphosate exposure disrupted progesterone and estrogen secretion, modified ovarian morphology, and altered the expression of steroidogenesis-related genes, with these changes frequently associated with oxidative stress [14]. Beyond its impact on sex hormones, glyphosate may also compromise sperm quality, mediated through heightened oxidative stress. A laboratory investigation involving sperm samples from 66 healthy men demonstrated that exposure to GBHs at a concentration of 1 mg/L significantly reduced sperm motility and impaired mitochondrial function [34].

Epidemiological studies link glyphosate exposure to oxidative stress markers. Occupational research shows that agricultural workers exhibit elevated 8-hydroxy-2′-deoxyguanosine and malondialdehyde [35,36]. In 227 pregnant U.S. women, higher urinary aminomethylphosphonic acid, a glyphosate metabolite, correlated with increased 8-iso-prostaglandin-F2α (Eaton et al., 2022b) [37]. A study of 128 infertile French men found that glyphosate exposure was tied to increased 8-hydroxy-2′-deoxyguanosine [38]. However, human epidemiological research examining the relationship between glyphosate and sex hormones remains scarce, predominantly relying on data from the NHANES. Furthermore, the scope of sex hormones investigated is narrow, typically limited to testosterone, estradiol, and SHBG. A study based on the 2013–2016 dataset found that urinary glyphosate in children and adolescents aged 6–19 was associated with reduced estradiol levels across the cohort, with a more pronounced effect observed in adolescents [15]. Another analysis from the 2013–2014 NHANES data, encompassing individuals aged 6–85, identified an inverse correlation between glyphosate levels and estradiol, along with a trend toward lower testosterone across all ages [16].

Our study expands available research by analyzing a wide range of sex hormone biomarkers, finding that higher urinary glyphosate levels correlate with reduced serum levels of AMH, androstenedione, estradiol, estrone, estrone sulfate, 17α-hydroxyprogesterone, and progesterone, while SHBG increases. The consistent negative associations observed between urinary glyphosate concentrations and serum levels of multiple sex hormones suggest that glyphosate may exert broad effects across the steroidogenesis pathway. As mentioned above, glyphosate has been shown in prior studies to inhibit aromatase activity, an enzyme critical for converting androgens (e.g., androstenedione) into estrogens (e.g., estradiol and estrone) [11]. This disruption could explain the observed reductions in estradiol, estrone, estrone sulfate, and the upstream precursor androstenedione. Similarly, glyphosate might affect other cytochrome P450 enzymes, such as CYP17A1, which catalyzes the production of 17α-hydroxyprogesterone and subsequent intermediates like progesterone, leading to their diminished levels [39,40]. Oxidative stress represents another plausible mechanism. Contact with glyphosate has been associated with heightened generation of reactive oxygen species and oxidative damage in experimental studies [33], which could impair mitochondrial function—a critical component of steroidogenesis. Mitochondria house CYP11A1, which initiates progesterone synthesis from cholesterol, and disruptions at this step could cascade to reduce downstream hormones such as 17α-hydroxyprogesterone, androstenedione, and estrogens [41].

The elevation in SHBG suggests that glyphosate’s capacity to diminish sex hormone precursors could reduce free sex hormone availability, potentially eliciting a compensatory rise in SHBG levels [42]. Importantly, SHBG is primarily synthesized in hepatocytes, while sex steroid hormones are produced mainly in the gonads. Thus, glyphosate may affect these biomarkers through distinct, tissue-specific mechanisms. In the liver, glyphosate may upregulate SHBG transcription via oxidative stress-mediated signaling pathways [11]. In contrast, within ovarian and testicular cells, glyphosate and its formulations have been shown to inhibit aromatase activity and increase oxidative stress, thereby disrupting normal steroidogenesis [41]. Recognizing these separate hepatic and gonadal pathways helps explain why we observed a positive association with SHBG alongside inverse associations with several sex hormones.

Typically, a decline in gonadal hormones like estradiol or testosterone would trigger compensatory increases in FSH and LH to stimulate steroid production. However, the stability of these gonadotropins in our study implies that glyphosate may not sufficiently alter the free fractions of these hormones to disrupt HPG feedback, possibly due to the concurrent SHBG elevation. Additionally, glyphosate’s potential to disrupt the HPG axis could also play a role. This hypothesis aligns with animal studies reporting glyphosate-induced changes in the HPG axis [13], though human evidence remains limited. Our findings highlight glyphosate’s potential as a multi-faceted endocrine disruptor, with implications for reproductive health that merit deeper mechanistic investigation.

In this investigation, age was observed to moderate the inverse association between glyphosate exposure and AMH concentrations, as indicated by a positive beta coefficient for the glyphosate–age interaction term, signifying a reduced effect with increasing age. AMH, an established indicator of Sertoli (in males) and granulosa (in females) cell activity that typically diminishes with age [20], exhibited a more substantial decline in younger participants with elevated baseline levels. Similarly, estrone, an estrogen predominantly synthesized in peripheral tissues, demonstrated an age-dependent reduction with glyphosate exposure, with the positive interaction term reflecting a less pronounced inverse relationship as age advances. These findings implied heightened susceptibility to glyphosate’s endocrine-disrupting properties during periods of maximal reproductive potential.

The gender-specific associations observed in this study—pronounced estrogen suppression in young males, broader steroidogenic impacts in adult males, and SHBG elevation in adult females—suggest that glyphosate’s endocrine-disrupting effects may vary due to sex-specific differences in gonadal physiology, steroidogenic enzyme activity, and hormonal regulation. Glyphosate is cleared rapidly in humans, with reported plasma half-lives of 3–4 h and urinary half-lives of 5–10 h, and >90% of an administered dose can be eliminated within 48 h [43]. Consequently, adipose accumulation is considered negligible. The lack of estrogen or androgen suppression in females may be attributed to protective antioxidant mechanisms in granulosa cells, which contrast with the more vulnerable male gonadal microenvironment [44]. These differential effects may also relate to sex-specific hormonal feedback loops, with males relying more on testicular steroidogenesis and females buffered by ovarian–pituitary interactions [45]. SHBG elevation in adult females could be explained by glyphosate possibly enhancing SHBG transcription in the liver through oxidative stress-mediated signaling, an effect potentially more pronounced in females due to the influence of elevated baseline estrogen on SHBG production [11,46]. Collectively, these mechanisms—oxidative stress, enzyme inhibition, and hormonal regulation—highlight glyphosate’s complex, sex-dependent endocrine disruption effect, necessitating further mechanistic studies and longitudinal assessments to clarify reproductive health implications across genders and life stages.

Our findings raise important public health concerns by suggesting that even low-level environmental exposure to glyphosate—common in the general population—may disrupt a broad spectrum of sex hormones. Such disruptions, especially during sensitive developmental periods, could potentially affect reproductive health, puberty timing, and long-term endocrine function. The observed age- and sex-specific effects highlight vulnerable subpopulations that may be at greater risk. These results support the growing body of evidence that glyphosate and GBHs might function as endocrine-disrupting agents, underscoring the need for continued biomonitoring, stricter regulatory oversight, and public health strategies aimed at minimizing exposure.

This investigation offers several significant strengths that underscore its scientific robustness. It examines the relationship between urinary glyphosate levels and an extensive array of sex hormone biomarkers, drawing upon data from a nationally representative cohort of the U.S. population (NHANES 2017–2018). The application of high-precision analytical methodologies, coupled with statistical adjustments to account for the complex sampling design and covariates, substantially enhances the validity and reliability of the findings. Additionally, the investigation extends beyond linear associations by examining interaction effects and non-linear relationships, thereby providing a more nuanced understanding of potential effect modifiers and dose–response patterns. Despite its strengths, this study has several limitations. First, the cross-sectional design prevents us from inferring causality. Second, because both urinary glyphosate and the sex hormones analyzed have short biological half-lives, our results capture only recent exposure and acute endocrine responses; assessment of cumulative effects will require studies with repeated exposure and hormone measurements. Third, residual confounding from unmeasured factors may still influence the observed associations. Fourth, thw NHANES does not record menstrual cycle day, so intra-cycle variation in estradiol, progesterone, FSH, and LH could not be controlled; although we stratified by menopausal status, some nondifferential misclassification is likely and would bias estimates toward the null. Fifth, the small sample of participants younger than 18 years limits the precision of subgroup estimates in youths. Sixth, information on residential or occupational exposure sources (e.g., proximity to farmland or herbicide-related jobs) was unavailable in the public NHANES dataset, limiting our ability to account for these potential confounders. Seventh, this study did not include measurements of certain key androgenic hormones such as total testosterone, free testosterone, and DHEA, which may also play important roles in endocrine function and glyphosate-related effects. Eighth, this exploratory study examined associations between urinary glyphosate and multiple sex hormones. While we used *p* < 0.05 as the significance threshold, we acknowledge the potential for type I error due to multiple comparisons. Given the biological interrelatedness of hormones, we did not apply overly conservative corrections that could increase type II error. Instead, we emphasized effect size, consistency, and biological plausibility. Several associations (e.g., androstenedione, estrone sulfate, SHBG) remained significant at *p* < 0.01, supporting their potential robustness. Finally, because the dataset represents only the U.S. population, caution is warranted when generalizing these findings to other geographic or cultural contexts.

## 5. Conclusions

In this nationally representative study, we found that higher urinary glyphosate levels were significantly associated with lower concentrations of multiple serum sex hormones, including AMH, androstenedione, estradiol, estrone, estrone sulfate, 17α-hydroxyprogesterone, and progesterone, while SHBG levels increased. These associations appeared to be linear and varied by age and sex, with stronger effects observed in males and in adults. Our findings extend previous research by demonstrating that glyphosate exposure may disrupt a broader range of sex hormones than previously recognized and may indicate endocrine-disrupting effects. However, while these associations are noteworthy, causality cannot be established due to the study’s cross-sectional design. Further longitudinal and mechanistic studies are warranted to confirm these associations and to better understand the potential reproductive and developmental health implications.

## Figures and Tables

**Table 1:** The mean (SD) of urinary glyphosate levels in different demographic subgroups.

	Number	Glyphosate (µg/g Creatinine)	*p* Value
Total	1532	0.38 (0.46)	
Sex			<0.001
Male	751	0.34 (0.36)	
Female	781	0.43 (0.54)	
Age (years)			<0.001
6–17	223	0.41 (0.38)	
18–59	842	0.31 (0.35)	
≥60	467	0.50 (0.62)	
Ethnicity			<0.01
Mexican-American	241	0.38 (0.55)	
Other Hispanic	140	0.38 (0.42)	
Non-Hispanic white	553	0.43 (0.49)	
Non-Hispanic black	324	0.30 (0.30)	
Non-Hispanic Asian	191	0.39 (0.47)	
Other ethnicity	83	0.42 (0.47)	
BMI z-score			<0.001
<−0.39	517	0.45 (0.60)	
0.5	505	0.37 (0.41)	
≥0.5	510	0.33 (0.32)	
Smoking status *			<0.05
Non-smoker	795	0.40 (0.50)	
ETS	223	0.34 (0.41)	
Current smoker	291	0.34 (0.43)	
Alcohol consumption (drink/year) *			<0.001
<12	540	0.33 (0.37)	
≥12	769	0.41 (0.52)	
eGFR (mL/min/1.73 m^2^) *			0.185
<60	70	0.45 (0.45)	
≥60	1239	0.37 (0.47)	

*p* values were calculated for differences in urinary glyphosate concentrations across demographic subgroups using Student’s *t*-test for binary variables (e.g., sex, smoking status) and one-way analysis of variance (ANOVA) for variables with more than two categories (e.g., age group, ethnicity, BMI z-score, and alcohol consumption); Abbreviations: eGFR, estimated glomerular filtration rate; ETS, environmental tobacco smoke; * for participants aged 18 and older; *p* values are shown to three decimal places when ≥0.05; significant *p* values are expressed as *p* < 0.05, *p* < 0.01 or *p* < 0.001.

**Table 2:** The median and interquartile range (25th–75th percentile) of urinary glyphosate and sex hormones in the studied population, stratified by age group (children, adolescents, and adults), with adult females further categorized into pre- and post-menopausal groups (N = 1532).

	Female				Male		
	Children	Adolescents	Pre-Menopause	Menopause	Children	Adolescents	Adults
Number	28	99	259	395	11	85	655
Glyphosate (ug/L)	0.60 (0.21–1.04)	0.26 (0.12–0.54)	0.22 (0.11–0.44)	0.23 (0.10–0.46)	0.38 (0.23–0.63)	0.38 (0.21–0.72)	0.28 (0.14–0.53)
Glyphosate (μg/g creatinine)	0.71 (0.52–1.45)	0.24 (0.17–0.45)	0.22 (0.13–0.36)	0.29 (0.17–0.55)	0.45 (0.29–0.62)	0.25 (0.14–0.37)	0.23 (0.13–0.38)
FSH (mIU/mL)	2.07 (1.19–3.31)	5.08 (3.87–6.63)	5.27 (3.55–7.20)	50.70 (31.31–73.45)	1.84 (0.44–2.36)	3.38 (2.24–5.11)	4.70 (3.11–7.93)
LH (mIU/mL)	0.10 (0.07–0.23)	5.39 (3.19–8.35)	7.48 (5.07–11.51)	26.78 (15.77–37.55)	0.60 (0.07–2.44)	4.08 (2.72–5.64)	5.77 (4.07–8.01)
AMH (ng/mL)	1.99 (1.58–2.66)	2.69 (1.92–4.41)	2.12 (0.77–4.07)	0.02 (0.02–0.02)	15.97 (11.28–37.28)	6.11 (4.42–8.48)	4.26 (2.67–6.78)
Androstenedione (ng/dL)	15.00 (8.13–22.03)	103.00 (72.50–147.00)	119.00 (83.50–151.00)	46.50 (32.40–67.10)	27.10 (12.80–32.90)	56.70 (40.55–76.00)	65.00 (49.70–85.60)
Estradiol (pg/mL)	1.91 (1.22–9.57)	49.70 (22.90–86.60)	101.00 (50.60–168.00)	6.78 (3.54–13.70)	1.85 (1.22–5.04)	18.40 (11.50–26.10)	24.10 (18.50–31.40)
Estrone (ng/dL)	0.42 (0.17–0.57)	3.94 (2.64–6.52)	7.59 (5.20–11.30)	2.53 (1.68–3.78)	0.57 (0.36–1.06)	2.09 (1.70–2.96)	3.42 (2.67–4.50)
Estrone Sulfate (pg/mL)	48.70 (25.43–77.45)	844.00 (376.00–1660.00)	1400.00 (744.00–2440.00)	262.00 (136.00–481.00)	61.20 (24.60–116.00)	452.00 (238.50–713.50)	615.00 (371.00–954.00)
17α-hydroxyprogesterone (ng/dL) *		35.95 (25.28–75.38)	69.30 (33.40–140.00)	17.30 (11.00–26.30)		70.90 (42.90–115.50)	65.60 (44.50–94.30)
Progesterone (ng/dL)	3.16 (1.97–3.86)	8.01 (5.01–84.80)	32.50 (7.28–743.00)	3.18 (2.01–5.50)	2.82 (1.93–5.03)	4.42 (2.97–9.09)	4.97 (3.21–7.89)
SHBG (nmol/L)	84.84 (61.54–118.80)	41.82 (27.69–57.74)	52.44 (34.62–78.63	50.54 (33.72–78.19)	89.39 (57.46–97.65)	26.26 (18.26–36.28)	34.68 (24.03–47.85)

Abbreviations: AMH: anti-Mullerian hormone; FSH: follicle-stimulating hormone; LH: luteinizing hormone; SHBG: sex hormone-binding globulin. * N = 83 (female adolescents), N = 399 (menopause), and N = 74 (male adolescents).

**Table 3:** Linear regression coefficients (S.E.) in ln-transformed serum sex hormone levels with a one-unit increase in ln-transformed urinary glyphosate concentrations, with results weighted for the sampling strategy.

	Glyphosate (µg/L)					
	β Coeff (S.E.)	*p* Value	*p* for Interaction (Age)	*p* for Interaction (Sex)	*p* for Interaction (Ethnicity)	*p* for Interaction (BMI z-Score)
FSH (mIU/mL)	0.083 (0.054)	0.144	0.138	0.843	0.505	0.715
LH (mIU/mL)	−0.024 (0.066)	0.721	0.896	0.217	0.070	0.168
AMH (ng/mL)	−0.140 (0.061)	<0.05	<0.05	0.958	0.305	0.338
Androstenedione (ng/dL)	−0.134 (0.027)	<0.001	0.879	0.686	0.813	0.661
Estradiol (pg/mL)	−0.185 (0.079)	<0.05	0.066	0.369	0.783	0.888
Estrone (ng/dL)	−0.132 (0.052)	<0.05	<0.05	0.110	0.932	0.874
Estrone Sulfate (pg/mL)	−0.196 (0.045)	<0.001	0.076	0.094	0.587	0.763
17α-hydroxyprogesterone (ng/dL) *	−0.097 (0.45)	<0.05	0.064	0.093	0.907	0.751
Progesterone (ng/dL)	−0.212 (0.085)	<0.05	0.835	0.744	0.519	0.378
SHBG (nmol/L)	0.080 (0.022)	<0.01	0.110	0.443	0.686	0.378

* N = 1464. Adjusted for Model 1: age, sex, race/ethnicity, BMI z-score, and urinary creatinine. Abbreviations: AMH: anti-Mullerian hormone; FSH: follicle-stimulating hormone; LH: luteinizing hormone; SHBG: sex hormone-binding globulin. *p* values are shown to three decimal places when ≥0.05; significant *p* values are expressed as *p* < 0.05, *p* < 0.01, or *p* < 0.001.

**Table 4:** Linear regression coefficients (S.E.) in ln-transformed serum sex hormone levels with a one-unit increase in ln-transformed urinary glyphosate concentrations in individuals aged under 18 years of age, stratified by sex, with results weighted for the sampling strategy.

	Glyphosate (µg/L)					
	Total		Male		Female	
Number	223		96		127	
	β Coefficient (S.E.)	*p* Value	β Coefficient (S.E.)	*p* Value	β Coefficient (S.E.)	*p* Value
FSH (mIU/mL)	−0.037 (0.048)	0.450	−0.135 (0.078)	0.105	0.008 (0.096)	0.937
LH (mIU/mL)	−0.134 (0.107)	0.227	−0.131 (0.089)	0.163	−0.196 (0.138)	0.177
AMH (ng/mL)	−0.022 (0.071)	0.763	0.097 (0.118)	0.425	−0.092 (0.058)	0.134
Androstenedione (ng/dL)	−0.075 (0.050)	0.154	−0.081 (0.068)	0.252	−0.096 (0.074)	0.215
Estradiol (pg/mL)	−0.103 (0.130)	0.440	−0.262 (0.095)	<0.05	−0.002 (0.198)	0.993
Estrone (ng/dL)	−0.056 (0.073)	0.454	−0.040 (0.057)	0.498	−0.094 (0.110)	0.408
Estrone sulfate (pg/mL)	−0.156 (0.096)	0.125	−0.374 (0.105)	<0.01	−0.075 (0.138)	0.593
17α-hydroxyprogesterone (ng/dL)	−0.030 (0.124)	0.815	−0.195 (0.199)	0.345	0.331 (0.180)	0.088
Progesterone (ng/dL)	0.149 (0.161)	0.371	−0.002 (0.158)	0.990	0.484 (0.270)	0.095
SHBG (nmol/L)	0.072 (0.085)	0.410	0.160 (0.074)	<0.05	−0.004 (0.066)	0.950

Adjusted for Model 1: age, sex, ethnicity, BMI z-score, and urinary creatinine. Abbreviations: AMH: anti-Mullerian hormone; FSH: follicle-stimulating hormone; LH: luteinizing hormone; SHBG: sex hormone-binding globulin. *p* values are shown to three decimal places when ≥0.05; significant *p* values are expressed as *p* < 0.05, *p* < 0.01, or *p* < 0.001.

**Table 5:** Linear regression coefficients (S.E.) in ln-transformed serum sex hormone levels with a one-unit increase in ln-transformed urinary glyphosate concentrations in individuals aged 18 years and older, stratified by sex, with results weighted for the sampling strategy.

	Glyphosate (µg/L)					
	Total		Male		Female	
Number	1309		655		654	
	β Coefficient (S.E.)	*p* Value	β Coefficient (S.E.)	*p* Value	β Coefficient (S.E.)	*p* Value
FSH (mIU/mL)						
Model 1	0.078 (0.056)	0.182	0.110 (0.055)	0.066	0.006 (0.078)	0.936
Model 2	0.074 (0.059)	0.228	0.104 (0.057)	0.090	0.023 (0.080)	0.779
LH (mIU/mL)						
Model 1	0.068 (0.056)	0.246	0.131 (0.068)	0.075	−0.018 (0.067)	0.789
Model 2	0.062 (0.062)	0.332	0.121 (0.071)	0.110	−0.011 (0.073)	0.883
AMH (ng/mL)						
Model 1	−0.115 (0.065)	0.098	−0.044 (0.055)	0.428	−0.048 (0.084)	0.573
Model 2	−0.103 (0.066)	0.140	−0.033 (0.049)	0.503	−0.066 (0.089)	0.469
Androstenedione (ng/dL)						
Model 1	−0.064 (0.028)	<0.05	−0.062 (0.032)	0.073	−0.045 (0.042)	0.294
Model 2	−0.069 (0.029)	<0.05	−0.081 (0.031)	<0.05	−0.052 (0.042)	0.235
Estradiol (pg/mL)						
Model 1	−0.064 (0.082)	0.446	−0.068 (0.025)	<0.05	0.008 (0.115)	0.943
Model 2	−0.058 (0.082)	0.489	−0.067 (0.024)	<0.05	0.007 (0.113)	0.952
Estrone (ng/dL)						
Model 1	−0.035 (0.047)	0.476	−0.084 (0.029)	<0.05	0.036 (0.067)	0.593
Model 2	−0.032 (0.047)	0.507	−0.088 (0.028)	<0.01	0.038 (0.065)	0.570
Estrone sulfate (pg/mL)						
Model 1	−0.075 (0.042)	0.092	−0.115 (0.032)	<0.01	−0.001 (0.069)	0.993
Model 2	−0.059 (0.043)	0.187	−0.094 (0.033)	<0.05	0.005 (0.069)	0.943
17α-hydroxyprogesterone (ng/dL)						
Model 1	−0.088 (0.041)	<0.05	−0.126 (0.035)	<0.01	−0.033 (0.064)	0.617
Model 2	−0.088 (0.040)	<0.05	−0.131 (0.036)	<0.01	−0.046 (0.063)	0.476
Progesterone (ng/dL)						
Model 1	−0.146 (0.082)	0.097	−0.087 (0.051)	0.109	−0.126 (0.136)	0.367
Model 2	−0.145 (0.084)	0.104	−0.100 (0.052)	0.071	−0.140 (0.133)	0.311
SHBG (nmol/L)						
Model 1	0.056 (0.021)	<0.05	0.046 (0.033)	0.182	0.072 (0.031)	<0.05
Model 2	0.056 (0.020)	<0.05	0.028 (0.029)	0.352	0.073 (0.032)	<0.05

Model 1 adjusted for age, sex, ethnicity, BMI z-score, and urinary creatinine. Model 2 adjusted for Model 1 plus smoking status, drinking status, and physical activity. Abbreviations: AMH: anti-Mullerian hormone; FSH: follicle-stimulating hormone; LH: luteinizing hormone; SHBG: sex hormone-binding globulin. *p* values are shown to three decimal places when ≥0.05; significant *p* values are expressed as *p* < 0.05, *p* < 0.01, or *p* < 0.001.

## Data Availability

The datasets analyzed in this study can be accessed on the NHANES website (https://wwwn.cdc.gov/nchs/nhanes/continuousnhanes/default.aspx?BeginYear=2017) (accessed on 5 June 2025).

## Supplementary Materials

The following supporting information can be downloaded at: https://www.mdpi.com/article/10.3390/life15071024/s1, Table S1. Mean (SD) of serum sex hormones in participants stratified by undetectable/detectable urinary glyphosate; Table S2. Linear regression coefficients (S.E.) in ln-serum sex hormone levels with a one-unit increase in ln-urinary glyphosate concentrations under 18 years of age, stratified by age group (children, adolescents), with results weighted for the sampling strategy. Table S3. Linear regression coefficients (S.E.) in ln-serum sex hormone levels with a one-unit increase in ln-urinary glyphosate concentrations in females aged 18 years and older, stratified by menopausal status, with results weighted for the sampling strategy. Table S4. Linear regression coefficients (S.E.) in ln-serum sex hormone levels with a one-unit increase in ln-urinary glyphosate concentrations, with results weighted for the sampling strategy. (N = 1528).

## Abbreviations

AMH	Anti-Müllerian hormone
BMI	Body mass index
eGFR	Estimated glomerular filtration rate
ETS	Environmental tobacco smoke
FSH	Follicle-stimulating hormone
GBH	Glyphosate-based herbicide
HPG	Hypothalamic–pituitary–gonadal
hs-CRP	High-sensitivity C-reactive protein
IC-MS/MS	Ion chromatography paired with tandem mass spectrometry
LH	Luteinizing hormone
LOD	Limit of detection
Ln	Natural logarithm
NHANES	National Health and Nutrition Examination Survey
SHBG	Sex hormone-binding globulin
