# Supplementary material for: Urinary Glyphosate Concentrations and Serum Sex Hormones in a Nationally Representative U.S. Sample: NHANES 2017–2018
Source: Life (Basel). 2025 Jun 27;15(7):1024. doi: 10.3390/life15071024 (PMC12298975; doi:10.3390/life15071024)
Supplement: Supplementary file 1 [file life-15-01024-s001.zip › life-3628869-supplementary.pdf]

Supplementary Material

Supplemental Table S1. Mean (SD) of serum sex hormones in participants stratified by undetectable/detectable urinary glyphosate

	Glyphosate (µg/L)		
	Undetectable (N=290)	Detectable (N=1255)	
	Mean (SD)	Mean (SD)	P value
FSH (mIU/mL)	21.63 (29.67)	17.81 (25.86)	< 0.05
LH (mIU/mL)	13.89 (14.47)	12.22 (13.19)	0.085
AMH (ng/mL)	3.45 (4.53)	3.72 (4.44)	0.185
Androstenedione (ng/dL)	82.14 (50.92)	76.74 (48.95)	0.092
Estradiol (pg/mL)	45.93 (79.03)	51.10 (247.89)	0.717
Estrone (ng/dL)	4.52 (3.87)	4.85 (11.10)	0.608
Estrone Sulfate (pg/mL)	950.14 (1235.88)	874.06 (1445.85)	0.417
17α-hydroxyprogesterone (ng/dL)*	66.56 (57.34)	64.60 (59.23)	0.607
Progesterone (ng/dL)	101.11 (341.43)	88.38 (407.32)	0.636
SHBG (nmol/L)	52.63 (35.32)	50.18 (37.62)	0.282

*N = 284 (undetectable), N = 1180 (detectable). p values are shown to three decimal places when ≥ 0.05; significant p values are expressed as $p < 0.05$, $p < 0.01$ or $p < 0.001$.

Supplemental Table S2. Linear regression coefficients (S.E.) in ln-serum sex hormone levels with a one-unit increase in ln-urinary glyphosate concentrations under 18 years of age, stratified by age group (children, adolescents), with results weighted for the sampling strategy.

	Glyphosate (µg/L)			
	Children		Adolescents	
Number	39		184	
	β coeff (S.E.)	P value	β coeff (S.E.)	P value
FSH (mIU/mL)	0.230 (0.92)	0.054	-0.075 (0.073)	0.323
LH (mIU/mL)	0.142 (0.098)	0.206	-0.108 (0.083)	0.212
AMH (ng/mL)	-0.220 (0.089)	0.056	0.055 (0.082)	0.509
Androstenedione (ng/dL)	0.022 (0.063)	0.745	-0.074 (0.053)	0.185
Estradiol (pg/mL)	0.300 (0.125)	0.061	-0.193 (0.128)	0.152
Estrone (ng/dL)	0.045 (0.058)	0.475	-0.052 (0.075)	0.500
Estrone Sulfate (pg/mL)	0.130 (0.150)	0.427	-0.230 (0.113)	0.059
17α-hydroxyprogesterone (ng/dL)			-0.030 (0.124)*	0.815
Progesterone (ng/dL)	0.013 (0.122)	0.920	0.176 (0.197)	0.385
SHBG (nmol/L)	0.129 (0.047)	< 0.05	0.119 (0.083)	0.171

Model 1 adjusted for age, sex, ethnicity, BMI z score, and urinary creatinine. Model 2 adjusted for Model 1 plus smoking status, drinking status, physical activity, and eGFR. Abbreviations: AMH: anti-Mullerian hormone; FSH: Follicle stimulating hormone; LH: Luteinizing hormone; SHBG: sex hormone binding globulin.

*N=156. p values are shown to three decimal places when ≥ 0.05; significant p values are expressed as $p < 0.05$, $p < 0.01$ or $p < 0.001$.

Supplemental Table S3. Linear regression coefficients (S.E.) in ln-serum sex hormone levels with a one-unit increase in ln-urinary glyphosate concentrations in females aged 18 years and older, stratified by menopausal status, with results weighted for the sampling strategy.

	Glyphosate (μg/L)			
	Pre-menopause		Menopause	
Number	259		395	
	β coeff (S.E.)	P value	β coeff (S.E.)	P value
FSH (mIU/mL)				
Model 1	-0.034 (0.099)	0.734	0.055 (0.082)	0.510
Model 2	-0.003 (0.101)	0.973	0.072 (0.073)	0.341
LH (mIU/mL)				
Model 1	-0.041 (0.130)	0.756	-0.013 (0.098)	0.898
Model 2	-0.012 (0.126)	0.926	-0.011 (0.098)	0.909
AMH (ng/mL)				
Model 1	0.024 (0.100)	0.809	-0.156 (0.093)	0.113
Model 2	0.028 (0.105)	0.794	-0.161 (0.077)	0.055
Androstenedione (ng/dL)				
Model 1	-0.030 (0.035)	0.402	-0.082 (0.050)	0.122
Model 2	-0.044 (0.039)	0.273	-0.085 (0.051)	0.113
Estradiol (pg/mL)				
Model 1	0.087 (0.103)	0.411	-0.134 (0.052)	0.022
Model 2	0.060 (0.102)	0.568	-0.130 (0.053)	0.028

Estrone (ng/dL)				
Model 1	0.121 (0.079)	0.148	-0.051 (0.051)	0.329
Model 2	0.091 (0.078)	0.262	-0.044 (0.047)	0.361
Estrone Sulfate (pg/mL)				
Model 1	0.135 (0.092)	0.163	-0.126 (0.071)	0.096
Model 2	0.095 (0.097)	0.341	-0.101 (0.071)	0.178
17α-hydroxyprogesterone (ng/dL)				
Model 1	-0.046 (0.050)	0.373	-0.079 (0.051)	0.139
Model 2	-0.073 (0.047)	0.139	-0.096 (0.050)	0.076
Progesterone (ng/dL)				
Model 1	-0.150 (0.177)	0.408	-0.206 (0.061)	0.004
Model 2	-0.219 (0.175)	0.230	-0.206 (0.057)	0.003
SHBG (nmol/L)				
Model 1	0.112 (0.062)	0.093	0.050 (0.035)	0.170
Model 2	0.104 (0.061)	0.109	0.048 (0.038)	0.228

Model 1 adjusted for age, sex, ethnicity, BMI z score, and urinary creatinine. Model 2 adjusted for Model 1 plus smoking status, drinking status, physical activity, and eGFR. Abbreviations: AMH: anti-Mullerian hormone; FSH: Follicle stimulating hormone; LH: Luteinizing hormone; SHBG: sex hormone binding globulin. p values are shown to three decimal places when ≥ 0.05; significant p values are expressed as $p < 0.05$, $p < 0.01$ or $p < 0.001$.

Supplemental Table S4. Linear regression coefficients (S.E.) in ln-serum sex hormone levels with a one-unit increase in ln-urinary glyphosate concentrations, with results weighted for the sampling strategy. (N=1528)

	Glyphosate (µg/L)	
	β coeff (S.E.)	p value
FSH (mIU/mL)	0.079 (0.053)	0.158
LH (mIU/mL)	-0.018 (0.066)	0.786
AMH (ng/mL)	-0.138 (0.061)	<0.05
Androstenedione (ng/dL)	-0.126 (0.028)	<0.001
Estradiol (pg/mL)	-0.175 (0.079)	<0.05
Estrone (ng/dL)	-0.118 (0.051)	<0.05
Estrone Sulfate (pg/mL)	-0.183 (0.045)	<0.001
17α-hydroxyprogesterone (ng/dL)*	-0.098 (0.46)	<0.05
Progesterone (ng/dL)	-0.209 (0.084)	<0.05
SHBG (nmol/L)	0.075 (0.021)	<0.01

* N=1463; Adjusted for Model plus hs-CRP; Abbreviations: AMH: anti-Mullerian hormone; FSH: Follicle stimulating hormone; LH: Luteinizing hormone; SHBG: sex hormone binding globulin. p values are shown to three decimal places when ≥ 0.05; significant p values are expressed as $p < 0.05$, $p < 0.01$ or $p < 0.001$.
